# Otopathogenic *Pseudomonas aeruginosa* Enters and Survives Inside Macrophages

**DOI:** 10.3389/fmicb.2016.01828

**Published:** 2016-11-18

**Authors:** Rahul Mittal, Christopher V. Lisi, Hansi Kumari, M’hamed Grati, Patricia Blackwelder, Denise Yan, Chaitanya Jain, Kalai Mathee, Paulo H. Weckwerth, Xue Z. Liu

**Affiliations:** ^1^Department of Otolaryngology, Miller School of Medicine, University of Miami, MiamiFL, USA; ^2^Department of Human and Molecular Genetics, Herbert Wertheim College of Medicine, Florida International University, MiamiFL, USA; ^3^Chemistry Department, Center for Advanced Microscopy, University of Miami, Coral GablesFL, USA; ^4^Rosenstiel School of Marine and Atmospheric Science, University of Miami, Key BiscayneFL, USA; ^5^Department of Biochemistry and Molecular Biology, Miller School of Medicine, University of Miami, MiamiFL, USA; ^6^Global Health Consortium and Biomolecular Science Institute, Florida International University, MiamiFL, USA; ^7^Health Sciences Department, University of Sagrado CoraçãoBauru, Brazil

**Keywords:** otopathogenic *P. aeruginosa*, monocyte-derived macrophages, mouse bone marrow-derived macrophages, cell viability

## Abstract

Otitis media (OM) is a broad term describing a group of infectious and inflammatory disorders of the middle ear. Despite antibiotic therapy, acute OM can progress to chronic suppurative otitis media (CSOM) characterized by ear drum perforation and purulent discharge. *Pseudomonas aeruginosa* is the most common pathogen associated with CSOM. Although, macrophages play an important role in innate immune responses but their role in the pathogenesis of *P. aeruginosa-*induced CSOM is not known. The objective of this study is to examine the interaction of *P. aeruginosa* with primary macrophages. We observed that *P. aeruginosa* enters and multiplies inside human and mouse primary macrophages. This bacterial entry in macrophages requires both microtubule and actin dependent processes. Transmission electron microscopy demonstrated that *P. aeruginosa* was present in membrane bound vesicles inside macrophages. Interestingly, deletion of *oprF* expression in *P. aeruginosa* abrogates its ability to survive inside macrophages. Our results suggest that otopathogenic *P. aeruginosa* entry and survival inside macrophages is OprF-dependent. The survival of bacteria inside macrophages will lead to evasion of killing and this lack of pathogen clearance by phagocytes contributes to the persistence of infection in CSOM. Understanding host–pathogen interaction will provide novel avenues to design effective treatment modalities against OM.

## Introduction

Innate immune system serves as the first-line of defense against invading pathogens during infection (Alberts et al., 2002; Clarke, 2014). One of the principal components of the innate immune system are macrophages (Tam and Aderem, 2014; Divangahi et al., 2015; Hume, 2015; Schultze et al., 2015). They are considered as “professional phagocytes” that play a crucial role in eradication of pathogens through phagocytosis leading to clearance of infection (Aderem, 2003). Stimuli like infections lead to activation of macrophages. In addition, they can rapidly attract neutrophils, monocytes and other immune cells from the blood and hematopoietic tissues to the site of infection by the release of cytokines and chemotactic substances (Zhang and Wang, 2014). This leads to an efficient orchestration of both innate and adaptive host immune responses. Given the prominent role of macrophages as an effector cell type in the host immune responses, it is not surprising that certain pathogens have evolved strategies to evade killing and use macrophages as a shield against cell-mediated and humoral immune responses to cause infection in humans. Some pathogens inhibit phagocytosis and subsequently activation of macrophages (Fällman et al., 2002). However, no information is available regarding host–pathogen interplay in the pathogenesis of otitis media (OM).

Otitis media refers to any inflammatory or infectious process involving the middle ear (Cunningham et al., 2012; Minovi and Dazert, 2014; Atkinson et al., 2015; Wallis et al., 2015). Chronic suppurative OM (CSOM) is a chronic inflammation of the middle ear characterized by persistent middle ear drainage through the perforated tympanic membrane for more than 6 weeks (Bluestone, 1998; Qureishi et al., 2014; Mittal et al., 2015). The most common cause of CSOM is the bacterial infection (Meyerhoff, 1988; Mittal et al., 2015). *Pseudomonas aeruginosa* is one of the leading causes of CSOM (Yeo et al., 2007; Dayasena et al., 2011; Madana et al., 2011; Afolabi et al., 2012). CSOM is one of the largest public health burdens worldwide leading to hearing loss and life-threatening central nervous system complications, including brain abscess and meningitis (Chew et al., 2012; Yorgancılar et al., 2013; Sun and Sun, 2014). There is an urgent need to develop new therapies to combat this disease and help prevent complications associated with it. Understanding the role of host immunity in the pathogenesis of CSOM will open up novel avenues of treatment against the disease other than antibiotics.

Macrophages are an integral component of innate immunity and provide efficient protection against pathogens; however, the role of macrophages in CSOM has not been elucidated. In this study, we characterized the interaction of otopathogenic *P. aeruginosa* with primary human monocyte-derived macrophages (MDMs) and mouse bone marrow-derived macrophages (BMMϕ), *in vitro*. Our data suggests that otopathogenic *P. aeruginosa* enters and survives inside human MDMs and mouse BMMϕ. Since bacterial outer membrane proteins (OMPs) play a crucial role in interaction of pathogens with immune cells (Confer and Ayalew, 2013), we determined the role of *P. aeruginosa* OprF in bacterial survival inside macrophages. OprF is the major OMP of *P. aeruginosa* and has been demonstrated to play an important role in interaction of this pathogen with host cells (Nestorovich et al., 2006; Bouffartigues et al., 2012; Reusch, 2012; Mishra et al., 2015). We observed that survival of otopathogenic *P. aeruginosa* inside macrophages requires bacterial *oprF* expression. The ability of *P. aeruginosa* to survive inside macrophages enables it to escape killing by potent host immune responses and this lack of clearance by phagocytes contributes to the persistence of infection in CSOM.

## Materials and Methods

### Cell Culture

Mouse BMMϕ were generated by harvesting bone marrow cells from murine tibias and femurs as described earlier (Godek et al., 2006; Weischenfeldt and Porse, 2008). Briefly, bone marrow cells were flushed from mouse bones (C57 BL6), and then differentiated into a macrophage phenotype by incubating in complete DMEM (cDMEM, 10% heat inactivated fetal bovine serum (FBS, Hyclone^®^, Logan, UT, USA), 30% L-929 fibroblast conditioned medium, 1% penicillin–streptomycin (Gibco, Carlsbad, CA, USA), 0.01 M Hepes buffer, 1 mM sodium pyruvate, and 1% of a × 100 MEM non-essential amino acids solution (all from Sigma, St. Louis, MO, USA) in Dulbecco’s modified Eagle’s medium (DMEM, Mediatech, Herndon, VA, USA). Cells were differentiated for 7 days with media changes every 2 days. This study was carried out in accordance with the recommendations in the Guide for the Care and Use of Laboratory Animals of the National Institutes of Health (NIH). The protocol was approved by the Institutional Animal Care and Use Committee (IACUC) of the University of Miami.

Human MDMs were generated by separating monocytes from peripheral blood obtained from healthy blood donors, as previously described (Welin et al., 2008; Eklund et al., 2010; Meng et al., 2015; Shinzaki et al., 2016). Donors were between age groups of 25–50 years, of either sex and were free from blood-borne diseases. In brief, monocytes were obtained by layering blood onto a density gradient, followed by centrifugation and isolation of the monocyte/lymphocyte fraction. Cells were seeded in culture flasks and allowed to adhere for 2 h before non-adherent lymphocytes were washed away. The monocytes were allowed to differentiate into human MDMs for 5–8 days in DMEM (Gibco, Carlsbad, CA, USA) containing 25 mM Hepes, 100 U/ml penicillin, 100 μg/ml streptomycin and 10% active human serum (all Sigma, St. Louis, MO, USA). All cell cultures were incubated under 37°C, 5% CO_2_, 98% humidity. Human whole blood was obtained from commercial vendors including Interstate blood bank and followed NIH guidelines for the protection of human subjects including informed signed consent from all subjects by these vendors. All the information regarding human subjects was deidentified and investigators were having no access to any human subject information. The institutional review board (IRB) of the University of Miami approved the study protocol.

### Bacterial Strains

The clinical strains of *P. aeruginosa* (10 strains) isolated from CSOM patients attending University of Miami Hospital or Hospital for Rehabilitation of Craniofacial Anomalies of the University of São Paulo, Bauru, Brazil were used in this study. The patients exhibited persistent chronic purulent otorrhoea through perforated tympanic membrane for more than 6 weeks confirmed by otolaryngologic diagnosis. The isolation and identification of *P. aeruginosa* was performed using standard methods (MacFaddin, 1976; Forbes et al., 1998). The *oprF* mutant and complemented strain of *P. aeruginosa* was generated as described in previous studies (Woodruff and Hancock, 1989; Horton et al., 1990; Schweizer and Hoang, 1995; Rietsch et al., 2005; Balasubramanian et al., 2012; Yakhnina et al., 2015) (Supplementary Materials). Bacteria were grown overnight at 37°C in Luria broth (LB) (Teknova, Hollister, CA, USA) in a rotary shaker.

### Invasion Assays

Gentamicin protection assays were used to quantify the extent of human MDM and mouse BMMϕ invasion and survival by otopathogenic *P. aeruginosa*. Briefly, cells were infected with bacteria at 1:1, 5:1, 10:1, 25:1, 50:1, 100:1 multiplicity of infection (MOI) (bacteria:cell) for 1, 2, 4, and 6 h. After incubation, the cells were washed five times with warm RPMI medium (Corning, NY, USA) followed by addition of medium containing gentamicin (200 μg/ml) (Life Technologies, Carlsbad, CA, USA) and further incubated for 1 h at 37°C. All *P. aeruginosa* isolates we used in this study were sensitive to gentamicin at this concentration. The cells were washed three times with RPMI and then lysed with 1% saponin (Sigma, St. Louis, MO, USA) to release intracellular bacteria. Serial dilutions were then plated on blood agar plates and bacterial colonies were counted the next day. In some experiments, bacteria were pretreated with 20% human or mouse pooled serum or heat inactivated pooled serum and then used to infect human MDMs and mouse BMMϕ, respectively. In some experiments, bacteria were pretreated with monoclonal anti-OprF antibody (kindly provided by Dr. Hancock) and then used in the invasion assay. The monoclonal antibody (mAb) was specific to surface epitopes of OprF and was generated as described previously (Finnen et al., 1992; Rawling et al., 1995). To determine the effect of cytoskeletal inhibitors, macrophages were pretreated with different concentrations of cytochalasin D, vinblastine, nocodazole, or colchicine (all from Sigma, St. Louis, MO, USA) for 30 min before infecting with bacteria, and maintained in the medium for the entire infection period.

### Scanning Electron Microscopy (SEM)

Human MDMs and mouse BMMϕ were cultured on glass cover slips and were infected with bacteria for 30 min to 8 h. After incubation, the cells were washed five times with warm phosphate buffer saline (PBS) buffer (Sigma, St. Louis, MO, USA) to remove unbound bacteria and were then processed for scanning electron microscopy (SEM). Samples were fixed in 2% glutaraldehyde (Electron Microscopy Sciences, Hatfield, PA, USA) in PBS buffer followed by three changes of PBS buffer for 10 min each. The samples were then post-fixed in 1% osmium tetroxide (Electron Microscopy Sciences, Hatfield, PA, USA) in PBS buffer for 45 min and rinsed in three changes of PBS buffer for 10 min each. The samples were dehydrated in a graded series of ethanol, dried in hexamethyldisilazane (HMDS) (Electron Microscopy Sciences, Hatfield, PA, USA) and mounted on carbon adhesive tabs fixed to metal stubs. The samples were coated with palladium in a plasma sputter coater and viewed in a SEM (FEI, ESEM-FEG XL-30).

### Transmission Electron Microscopy (TEM)

Human MDMs and mouse BMMϕ were infected with bacteria for 30 min to 8 h. After incubation, the cells were washed with PBS and fixed using 2% glutaraldehyde (Electron Microscopy Sciences, Hatfield, PA, USA). The samples were rinsed in three washes of PBS buffer then post-fixed in 1% osmium tetroxide (Electron Microscopy Sciences, Hatfield, PA, USA) in 0.1 M phosphate buffer for 1 h. After buffer rinses, specimens were dehydrated through a series of graded ethanol, placed in two rinses of propylene oxide (Electron Microscopy Sciences, Hatfield, PA, USA) for 5 min each and then put in a 1:1 mixture of propylene oxide: EMbed/Araldite resin (Electron Microscopy Sciences, Hatfield, PA, USA) for overnight incubation at room temperature. Next day, the pellets were placed in fresh EMbed/Araldite and put in a vacuum desiccator for 2–4 h. The samples were changed to fresh EMbed/Araldite and polymerized overnight. Silver/gold sections were then cut on a Leica Ultracut E (Leica, Buffalo Grove, IL, USA), stained in uranyl acetate (Electron Microscopy Sciences, Hatfield, PA, USA) and lead citrate (Electron Microscopy Sciences, Hatfield, PA, USA), and viewed in a JEOL 1400 electron microscope (JEOL, Peabody, MA, USA) with Gatan Orius SC1000 camera (Gatan, Pleasanton, CA, USA).

### Immunofluorescence

For staining of bacteria and actin, human MDMs and mouse BMMϕ were cultured in 8-well chamber slides and infected with *P. aeruginosa* for 30 min to 8 h. After incubation, cells were washed three times with PBS buffer and then fixed and permeabilized with BD cytofix and cytoperm reagent (BD Biosciences, San Jose, CA, USA) for 30 min. After washing, the cells were blocked with 3% normal goat serum (NGS) (Life Technologies, Carlsbad, CA, USA) for 20 min and then incubated with anti-*P. aeruginosa* antibody (1:200) (Abcam, Cambridge, MA, USA) for 45 min followed by Alexa Fluor 488 antibody (1:1000) (Life Technologies, Carlsbad, CA, USA). After washing, cells were counterstained for actin with rhodamine phalloidin (Life Technologies, Carlsbad, CA, USA) for 45 min, washed and mounted in an antifade Vectashield solution containing 4, 6-diamidino-2-phenylindole (DAPI) (Vector Laboratories, Burlingame, CA, USA). The cells were viewed with a Zeiss LSM 710 microscope (Carl Zeiss, Germany) and images were assembled using Adobe photoshop 7.0.

### Cell Viability

To evaluate the cytotoxic effects of *P. aeruginosa* on human MDMs and mouse BMMϕ during the course of infection, cells were seeded in 96 well plates and infected with bacteria for 2–24 h at an MOI of 10. Lactate dehydrogenase (LDH) levels were then determined in cell culture supernatants using commercially available kit, as per the manufacturer’s instructions (Cayman Chemical, Ann Arbor, MI, USA). Uninfected cells were included as negative control. Maximum LDH release induced by treatment of macrophages with 1% Triton X-100 (Sigma, St. Louis, MO, USA) was used as a positive control. Results were expressed as percentage LDH release compared to the positive control.

The viability of *P. aeruginosa* infected human MDMs and mouse BMMϕ was also determined by ethidium homodimer-1 staining (Eidet et al., 2015; McCanna et al., 2015; Yoeruek et al., 2016). Ethidium homodimer-1 enters inside dead cells through the disrupted plasma membrane and binds to nucleic acids that increase its fluorescence intensity leading to the production of red fluorescence. Live cells exclude ethidium homodimer-I staining due to the intact plasma membrane. Human MDMs and mouse BMMϕ were infected with bacteria for 2–24 h time periods at an MOI of 10 and then stained with ethidium homodimer-1. Fluorescence was then determined using a microplate reader with appropriate filters (excitation at 535 nm and emission at 620 nm). The percentage dead cells were then calculated using the following formula:

$$\begin{array}{c} \%\mathrm{Dead}\mathrm{Cells}=[F{\left(620\mathrm{nm}\right)}_{\mathrm{sample}}-F{\left(620\mathrm{nm}\right)}_{\mathrm{minimum}}]\\ \;\times 100/[F{\left(620\mathrm{nm}\right)}_{\mathrm{maximum}}-F{\left(620\mathrm{nm}\right)}_{\mathrm{minimum}}] \end{array}$$

where *F*(620 nm) is fluorescence intensity at a wavelength of 620 nm, *F*(620 nm)_sample_ is fluorescence intensity of human MDMs and mouse BMMϕ infected with *P. aeruginosa, F*(620 nm)_minimum_ is fluorescence intensity of uninfected human MDMs and mouse BMMϕ, and *F*(620 nm)_maximum_ is fluorescence intensity of human MDMs and mouse BMMϕ treated with 1% Triton X-100 (Sigma, St. Louis, MO, USA).

### Statistical Analysis

Statistical significance was determined by a paired, two-tailed Student’s *t*-test and ANOVA using SPSS software. Values of *P* < 0.05 were considered to be statistically significant.

## Results

### Otopathogenic *P. aeruginosa* Enters and Survives Inside Primary Human MDMs and Mouse BMMϕ

**FIGURE 1:**
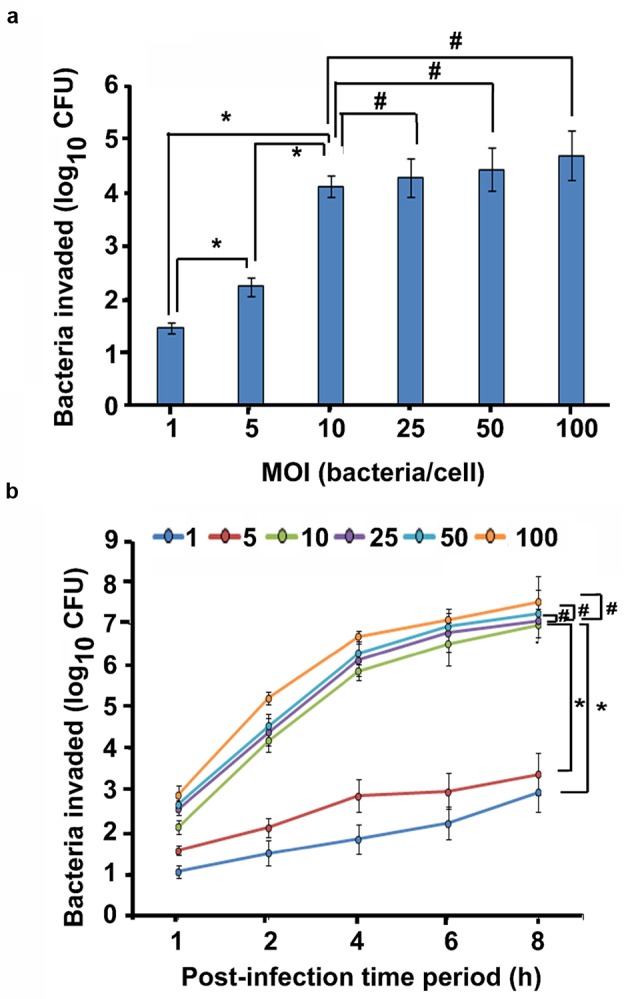
**Otopathogenic *Pseudomonas aeruginosa* enters and survives inside macrophages.** Human MDMs were infected with a clinical isolate of *P. aeruginosa* for 2 h and intracellular survival was determined by gentamicin protection assay **(a)**. In separate experiments, human MDMs were infected with *P. aeruginosa* at different MOIs for 1–8 h and phagocytosis was determined **(b)**. Data represents mean ± SD and is representative of five individual experiments carried out in triplicate. ^∗^*P* < 0.01 or #*P* > 0.05 by Student’s *t-*test and ANOVA.

Since *P. aeruginosa* is the major etiological agent of CSOM, we hypothesize that this pathogen hijacks host innate immunity that forms the first line of defense. To test this hypothesis, we examined the ability of a clinical isolate of *P. aeruginosa* from CSOM patient to enter and survive inside primary human and mouse macrophages by gentamicin protection assay at different MOIs for 2 h. We observed that *P. aeruginosa* is able to enter and survive inside macrophages. At an MOI of 1, log 1.45 colony forming unit (cfu) bacteria were recoverable from human MDMs whereas log 1.12 cfu bacteria were demonstrable inside mouse BMMϕ (**Figure 1a**). The number of bacteria inside human MDMs increased from 2.23 to 4.11 log cfu with a corresponding increase in MOI from 5 to 10 (**Figure 1a**). Similar increase in number of bacteria was observable inside mouse BMMϕ. The number of bacteria increased from log 2.11 log cfu at an MOI of 5 to log 3.48 cfu at an MOI of 10 (Supplementary Figure S1a). Further increase in MOI to 25, 50, and 100 caused only marginal increase in number of bacteria inside human MDMs and mouse BMMϕ (*P* > 0.05). Therefore, we selected a MOI of 10 and post-infection time period of 2 h for further experiments.

In order to determine the effect of increasing infection time on the cell invasion, macrophages and *P. aeruginosa* were incubated for several time-points. By 1 h post-infection time period, 1.04 log cfu bacteria were demonstrable inside human MDMs that increased to 2.96 log cfu bacteria by 8 h post-infection at an MOI of 1 (**Figure 1b**). At an MOI of 5 and 10, there were 1.56 and 2.12 log cfu bacteria at 1 h post-infection that increased to 3.39 and 6.98 at 8 h post-infection time period respectively. At a high MOI of 100, 7.55 log cfu bacteria were recoverable from human MDMs at 8 h post-infection time period. Mouse BMMϕ also demonstrated increase in bacterial load with the corresponding increase in post-infection time period from 1 to 8 h at all MOIs (Supplementary Figure S1b). Similar results of human MDM and mouse BMMϕ invasion was observed with nine additional *P. aeruginosa* CSOM clinical isolates at an MOI of 10 (Supplementary Figures S2 and S3). There was increase in bacterial load in both human MDMs and mouse BMMϕ with increase in post-infection time period from 2 to 6 h. Taken together, these results suggest that otopathogenic *P. aeruginosa* invades human MDMs and mouse BMMϕ in a time and dose dependent manner.

### Opsonization Has No Effect on the Invasion of Macrophages by *P. aeruginosa*

**FIGURE 2:**
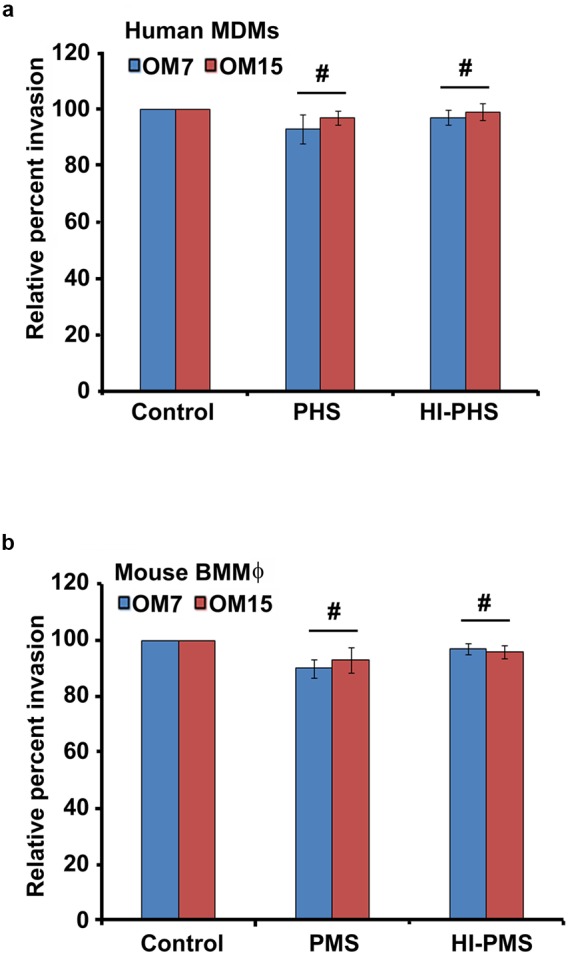
**Bacterial opsonization has no significant effect on internalization of *P. aeruginosa* by macrophages.** Otopathogenic *P. aeruginosa* isolates (OM7 and OM15) were pretreated with 20% pooled human serum (PHS) or pooled mouse serum (PMS) or heat-inactivated serum (HI-PHS or HI-PMS) or left untreated (control) and then used to infect human MDMs **(a)** and mouse BMMϕ **(b)**. The invasion of macrophages by bacteria was then determined by gentamicin protection assay. Results were expressed as percentage invasion relative to control group. Data represents mean ± SD and is representative of four individual experiments carried out in triplicate. #*P* > 0.05 compared to control by Student’s *t-*test and ANOVA.

Complement proteins are constitutively present in the serum and can opsonize bacteria non- specifically promoting pathogen killing and clearance of infection (Daha, 2010; Merle et al., 2015; Varela and Tomlinson, 2015). Therefore, we evaluated whether complement affects the phagocytosis of *P. aeruginosa* by macrophages. To determine the effect of opsonins on the survival of *P. aeruginosa*, two CSOM bacterial isolates were opsonized with fresh normal or heat-inactivated human or mouse pooled serum or were treated with medium alone prior to infection of human MDMs and mouse BMMϕ. Interestingly, there was no statistical difference in the number of non-opsonized and opsonized bacteria in human MDMs and mouse BMMϕ (*P* > 0.05; **Figures 2a,b**). The heat-inactivated serum also had no significant effect on the ability of otopathogenic *P. aeruginosa* to invade macrophages (*P* > 0.05). These results suggest that complement did not play a significant role in invasion of human MDMs and mouse BMMϕ by *P. aeruginosa*.

### Ultrastructural Examination of *P. aeruginosa* Infected Human MDMs and Mouse BMMϕ

**FIGURE 3:**
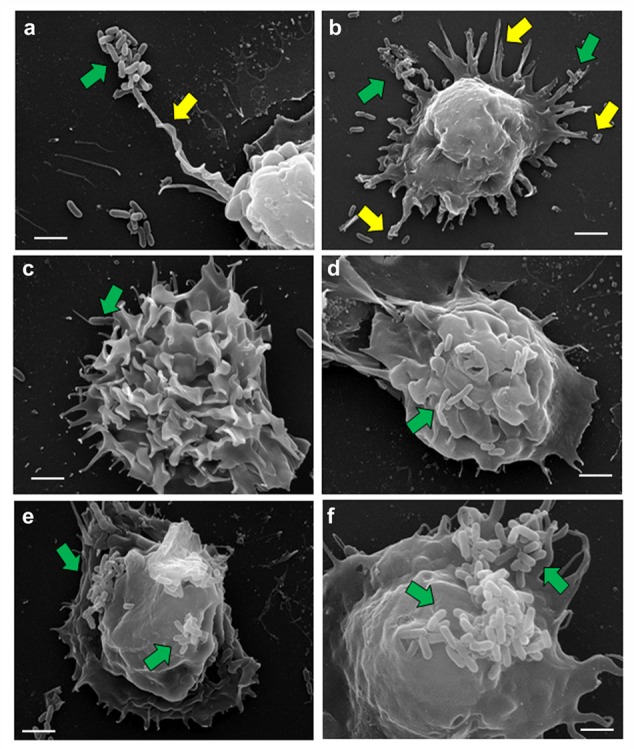
**Scanning electron micrographs of human MDMs infected with *P. aeruginosa*.** Human MDMs were infected with *P. aeruginosa* for 15 min **(a)**, 30 min **(b)**, 1 h **(c)**, 2 h **(d)**, 4 h **(e)**, and 8 h **(f)** and subjected to SEM. We observed that *P. aeruginosa* (green arrows) attach to pseudopod like structures (yellow arrows) on human macrophages. Results are representative of three individual experiments. Scale bars 2 μm.

To examine the interaction of *P. aeruginosa* with macrophages in detail, human MDMs and mouse BMMϕ were subjected to SEM. During the first 15 min of post-infection, *P. aeruginosa* was observed to adhere to human MDMs through pseudopod like structures (**Figure 3a**). There was an increase in the formation of these pseudopod like structures at 30 min post-infection (**Figure 3b**). Bacteria were seen to be adhered to the human MDMs mostly in clusters. However, individually attached bacteria were also observed. By 1 h post-infection, few bacteria were demonstrable on the surface of human MDMs accompanied with membrane ruﬄing (**Figure 3c**). The number of bacteria on the surface of human MDMs increased with increase in post-infection time period (**Figures 3d,e**). A large number of bacteria were observed on the surface of macrophages by 8 h post-infection (**Figure 3f**). Similarly few bacteria were demonstrable on the surface of mouse BMMϕ at 60 min post-infection that increased in number with increase in post-infection time period to 8 h. As observed with human MDMs, we also observed pseudopod formation and membrane ruﬄing in *P. aeruginosa* infected mouse BMMϕ (Supplementary Figure S4).

**FIGURE 4:**
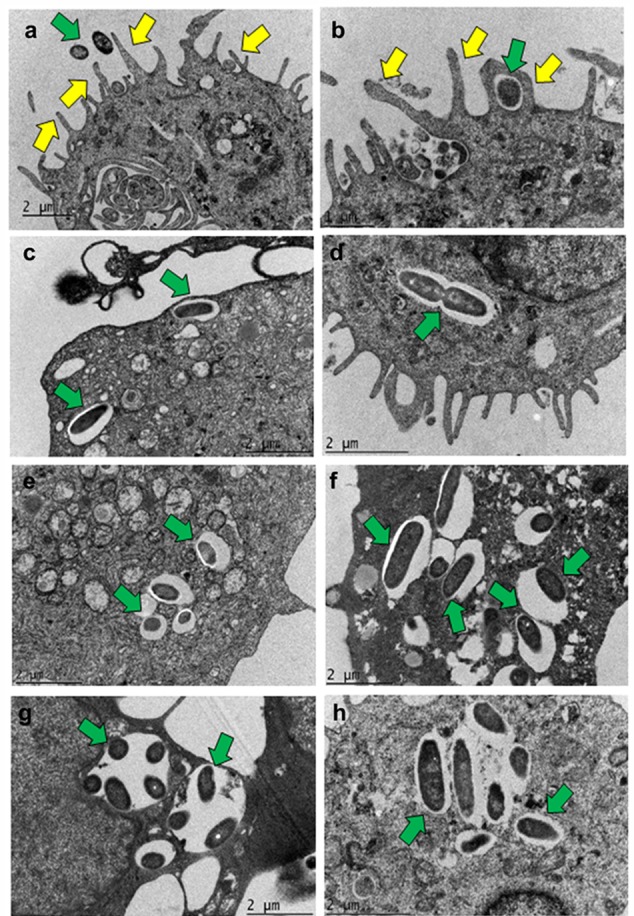
**Transmission electron micrographs demonstrating phagocytosis of *P. aeruginosa* by macrophages.** Human MDMs were infected with *P. aeruginosa* for 15 min **(a)**, 30 min **(b)**, 1 h **(c,d)**, 2 h **(e)**, 4 h **(f)**, and 8 h **(g,h)** and subjected to TEM. Bacteria were demonstrable inside membrane bound vacuoles in human MDMs which eventually were disrupted by 8 h post-infection. Yellow arrows indicate pseudopod like structures and green arrows indicate bacteria. Results are representative of three individual experiments. Scale bars **(a,c–h)** 2 μm; **(b)** 1 μm.

Transmission electron microscopy (TEM) demonstrated that human MDMs form plasma membrane protrusions in response to *P. aeruginosa* infection that can act as bacterial adhesion sites (**Figure 4a**). The bacteria were found in the enclosed membrane protrusions by 30 min post-infection in human MDMs (**Figure 4b**). At 60 min post-infection, *P. aeruginosa* was internalized into human MDMs inside membrane bound vacuoles (**Figure 4c**). In some vacuoles, the membrane was closely apposed to the bacteria whereas in others there was a space between bacteria and the membrane. In addition, actively dividing bacteria inside human MDMs were also observed (**Figure 4d**). There was increase in bacterial number inside human MDMs at 2 h post-infection (**Figure 4e**). At 4 h post-infection, the vacuoles containing bacteria increased in size (**Figure 4f**). At 8 h post-infection, there was clustering of bacteria inside human MDMs. In some vacuoles, multiple bacteria were demonstrable inside them (**Figure 4g**). At this post-infection time-period, *P. aeruginosa* disrupted the membrane and many free bacteria were observed in the cytoplasm of human MDMs (**Figure 4h**). Mouse BMMϕ displayed similar phenotypic changes in response to otopathogenic *P. aeruginosa* infection (Supplementary Figure S5).

### Entry of Otopathogenic *P. aeruginosa* Is Dependent on Both Microfilament and Microtubule Associated Pathway

**FIGURE 5:**
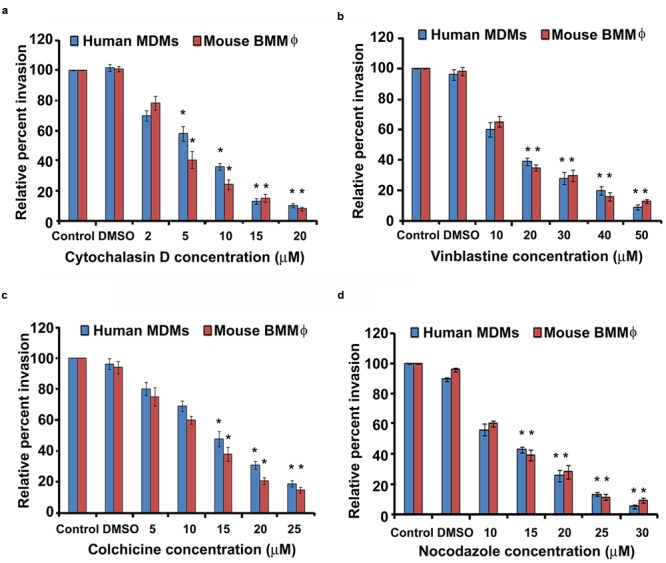
**Macrophages phagocytose *P. aeruginosa* through microfilament and microtubule dependent uptake mechanisms.** Human MDMs or mouse BMMϕ were pretreated with cytochalasin D **(a)**, vinblastine **(b)**, colchicine **(c)**, and nocodazole **(d)** and then infected with *P. aeruginosa*. Phagocytosis of *P. aeruginosa* by macrophages was determined by gentamicin protection assay. Data represents mean ± SD and is representative of four individual experiments carried out in triplicate. ^∗^*P* < 0.01 compared to control by Student’s *t-*test and ANOVA.

Host cytoskeleton can play a crucial role in bacterial cell invasion. Therefore, we determined whether invasion of macrophages by *P. aeruginosa* relies on microfilament and microtubule dependent pathways. Human MDMs and mouse BMMϕ were infected with *P. aeruginosa* in the presence of increasing concentrations of cytochalasin D, an inhibitor of actin polymerization. There was a significant dose-dependent decrease in the invasion of human MDMs and mouse BMMϕ in the presence of cytochalsin D compared to DMSO treated or untreated cells (*P* < 0.01) (**Figure 5a**). A 30% decrease in invasion in the presence of 2 μM cytochalasin D was observed whereas 90% decrease in invasion was observable at a concentration of 20 μM in human MDMs (**Figure 5a**). Microtubule disrupting compounds, vinblastine, colchicine, and nocodazole also caused a dose-dependent decrease in the invasion of human MDMs by *P. aeruginosa* (*P* < 0.01). Human MDMs pretreated with 10 μM vinblastine showed 40% decrease in invasion whereas cells pretreated with 50 μM demonstrated more than 90% decrease in invasion compared to DMSO treated or untreated macrophages (**Figure 5b**). Colchicine caused an 80% decreased in invasion at a concentration of 25 μM, whereas a 95% decrease in invasion was observed in the presence of 30 μM nocodazole (**Figures 5c,d**). Similar significant decrease in invasion of mouse BMMϕ was observed in the presence of cytochalsin D, vinblastine, colchicine, and nocodazole (*P* < 0.01) (**Figures 5a–d**). We observed that there were no toxic effects of these reagents on bacteria or on cells at the tested concentrations (data not shown). These results with inhibitory compounds suggest that *P. aeruginosa* invades human MDMs and mouse BMMϕ through both microfilament and microtubule dependent uptake mechanisms.

### *P. aeruginosa* Induces Actin Cytoskeleton Rearrangements during Invasion of Macrophages

**FIGURE 6:**
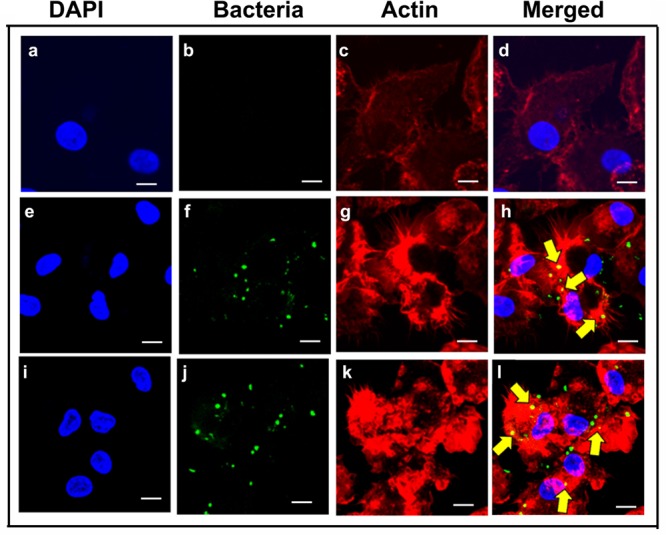
***Pseudomonas aeruginosa* induces actin cytoskeletal rearrangements during invasion of macrophages.** Human MDMs were infected with *P. aeruginosa* for 30 min **(e–h)** or 60 min **(i–l)** or left uninfected **(a–d)** and then stained with anti-*P. aeruginosa* antibody followed by Alexa Fluor 488 (green). Cells were counterstained with rhodamine phalloidin to visualize actin (red) and DAPI for cell nuclei (blue). Results are representative of three individual experiments. Scale bars 10 μm.

Actin cytoskeleton rearrangement is a common strategy employed by pathogens to invade host cells (Yang et al., 2012; Navarro-Garcia et al., 2013; de Souza Santos and Orth, 2015; Zheng et al., 2015). To determine whether *P. aeruginosa* induces alterations to the cytoskeleton during invasion of human MDMs and mouse BMMϕ, the distribution of F-actin in macrophages was examined. Human MDMs and mouse BMMϕ were infected with *P. aeruginosa* and stained with rhodamine phalloidin to detect F-actin. Uninfected human MDMs showed spatial distribution of F-actin throughout the cell (**Figures 6a–d**). However, infected human MDMs showed actin condensation in response to otopathogenic *P. aeruginosa* infection (**Figure 6**). At 30-min post-infection, a lot of actin accumulation underneath the bacterial binding sites was observed (**Figures 6e–h**). There was a lot of interaction of bacteria with the actin filaments. At 60-min, there was further increase in actin accumulation that colocalized with the bacteria as indicated by the yellow color (**Figures 6i–l**). Similar pattern of actin accumulation was observed in mouse BMMϕ infected with otopathogenic *P. aeruginosa* (data not shown). Taken together, these findings suggest that *P. aeruginosa* induces actin cytoskeleton rearrangements that facilitate its entry inside macrophages.

### Bacterial OprF Expression Plays a Crucial Role in Invasion of Macrophages by *P. aeruginosa*

**FIGURE 7:**
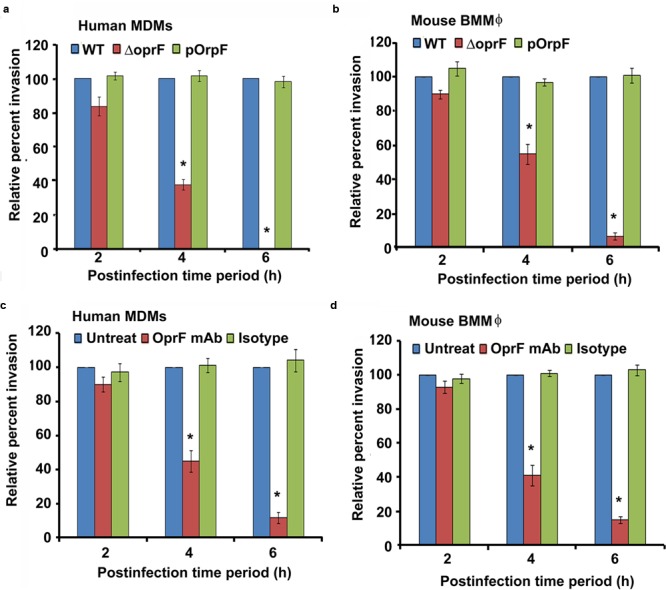
**Intracellular survival of *P. aeruginosa* inside macrophages requires bacterial *oprF* expression.** Human MDMs and mouse BMMϕ were infected with wild-type (WT), Δ*oprF* mutant or plasmid complemented (pOprF) strains of *P. aeruginosa*. Phagocytosis of bacteria by human MDMs **(a)** and mouse BMMϕ **(b)** was determined by gentamicin protection assay. In separate experiments, bacteria were pretreated with anti-oprF monoclonal antibody or left untreated and then used to infect human MDMs **(c)** and mouse BMMϕ **(d)**. Results were expressed as percentage compared to the phagocytosis of the WT strain. Data represents mean ± SD and is representative of four individual experiments carried out in triplicate. ^∗^*P* < 0.01 compared to control by Student’s *t-*test and ANOVA.

Bacterial OMPs play a crucial role in interaction of pathogens with host cells (Toma et al., 2014; Alzahrani et al., 2015; Bulir et al., 2015). OprF is the major OMP/porin of *P. aeruginosa* that has been demonstrated to facilitate biofilm formation under anaerobic conditions and adhesion to host cells (Azghani et al., 2002; Yoon et al., 2002). Therefore, we examined whether OprF play a role in the invasion of macrophages by *P. aeruginosa*. Both human and mouse macrophages were infected with wild-type (WT), Δ*oprF* and *trans-*complemented (pOprF) strains of *P. aeruginosa*. At 2 h post-infection, there was no significant difference (*P* > 0.05) in the phagocytosis of Δ*oprF* mutant strain of *P. aeruginosa* compared to WT or pOprF strains (**Figures 7a,b**). However, the number of Δ*oprF* bacterial mutant decreased significantly inside both human MDMs and mouse BMMϕ at 4 h post-infection (*P* < 0.01). At 6 h post-infection, no viable Δ*oprF* bacteria were recoverable from human MDMs and very low number of bacteria from mouse BMMϕ. In contrast, high numbers of bacteria were recoverable from WT or pOprF infected human MDMs and mouse BMMϕ.

To further confirm the role of OprF in invasion of macrophages by *P. aeruginosa*, WT bacteria were pretreated with anti-OprF monoclonal antibody or isotype control or left untreated and then used to infect human MDMs and mouse BMMϕ. Pretreatment of bacteria with anti-OprF monoclonal antibody significantly decreased the number of bacteria recoverable from human MDMs at 4 and 6 h post-infection compared to isotype control treated or untreated bacteria (*P* < 0.01) (**Figure 7c**). Similar decrease in number of intracellular *P. aeruginosa* was observed at 4 and 6 h post-infection when pretreated bacteria were used to infect mouse BMMϕ (**Figure 7d**). These results suggest that OprF plays a critical role in the survival of *P. aeruginosa* inside macrophages.

### Otopathogenic *P. aeruginosa* Exerts Cytopathic Effect on Macrophages

**FIGURE 8:**
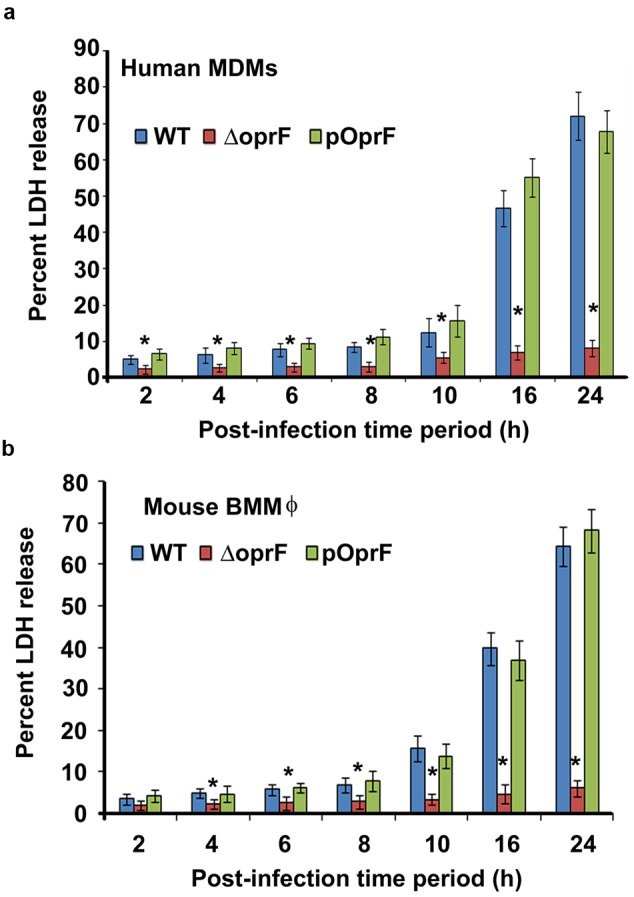
**Otopathogenic *P. aeruginosa* affect viability of macrophages.** Human MDMs **(a)** or mouse BMMϕ **(b)** were infected with *P. aeruginosa* at an MOI of 10 and viability of the macrophages was determined by measuring LDH levels in cell culture supernatants. Results were expressed as the percentage compared with maximum LDH release by lysed cells. Data represents mean ± SD and is representative of four individual experiments carried out in triplicate. ^∗^*P* < 0.01 compared to WT or pOprF by Student’s *t-*test and ANOVA.

To determine whether infection of macrophages with *P. aeruginosa* causes cell death. Human MDMs and mouse BMMϕ were infected with *P. aeruginosa* at an MOI of 10 and the levels of LDH released were determined in the cell culture supernatants. LDH release is the most acceptable and reliable marker to determine cell viability^34-36^. There was not much cell damage up to 8 h post-infection as demonstrated by minimal LDH release in cell culture supernatants of both human MDMs and mouse BMMϕ infected with WT otopathogenic *P. aeruginosa* (**Figures 8a,b**). However, a further increase in time resulted in significant cell death (*P* < 0.01). LDH levels increased from 12.5% at 10 h post-infection to 46.7% by 16 h post-infection in culture supernatants of infected human MDMs. Similarly, LDH levels increased from 15.7% at 8 h post-infection to 39.8% at 16 h post-infection in mouse BMMϕ. At 24 h post-infection, there was even a higher level of cell death, as indicated by high LDH levels in human MDMs and mouse BMMϕ. Interestingly, deletion of *oprF* abrogated the ability of otopathogenic to induce significant cell death in macrophages (*P* < 0.01). However, complementation with the pOprF plasmid restored the ability of mutant strain to exert cytopathic effects on human MDMs and mouse BMMϕ.

To further confirm that macrophages undergo apoptosis, infected human MDMs and mouse BMMϕ were stained with ethidium homodimer-1, a high affinity, membrane-impermeant dye that exclusively stains the DNA of dead cells (Eidet et al., 2015; McCanna et al., 2015; Yoeruek et al., 2016). In agreement with the LDH assay results, at 8 h post-infection very little ethidium homodimer-1 staining of infected human MDMs was observed (*P* > 0.05) (Supplementary Figure S6). However, 25% of human MDMs were stained with membrane-impermeant dye at 10 h post-infection. At 16 h post-infection, 50% of infected MDMs failed to exclude the dye. By 24 h post-infection, almost 75% of human MDMs had been stained with ethidium homodimer-1 suggesting considerable cell death. Similar results were obtained with mouse BMMϕ demonstrating extensive cell death at 24 h post-infection (Supplementary Figure S6). On par with our LDH data, human MDMs and mouse BMMϕ infected with Δ*oprF* mutant strain did not demonstrate significant ethidium homodimer-1 staining even at 24 h post-infection compared to WT infected cells (*P* < 0.01). This phenotype was restored upon complementation with pOprF. These results suggest that otopathogenic *P. aeruginosa* exerts cytopathic effects on macrophages for which bacterial OprF expression is necessary.

## Discussion

Despite advances in medical therapy, CSOM is still a clinically challenging disease. Antibiotics are the only available treatment modalities against CSOM at present but these have moderate efficacy against the disease, and at times are not effective at all. The antibiotics used to treat CSOM include neomycin, ciprofloxacin, cefepime, carbapenem, levofloxacin, and ceftazidime (Saunders J. et al., 2011; Mittal et al., 2015). However, *P. aeruginosa* has been demonstrated to be resistant to a wide variety of antibiotics including β lactams (penicillins, cephalosporins, and carbapenems), fluoroquinolones (ciprofloxacin), polymyxins and macrolides (erythromycin and azithromycin) (Nordmann and Guibert, 1998; Livermore, 2002; Walsh et al., 2003; Jang and Park, 2004; Poole, 2004, 2011; Saunders J. et al., 2011; Morita et al., 2014; Mittal et al., 2015). *P. aeruginosa* exhibits some degree of sensitivity to aminoglycosides but this class of antibiotics has significant ototoxicity and is not recommended for the treatment of CSOM (Black et al., 2004; Jing et al., 2015; Koo et al., 2015; Leis et al., 2015). A better knowledge of the interaction of pathogens with immune cells will provide new opportunities to design effective novel therapeutic strategies against CSOM. Although immune cells play an important role in clearance of infection, the interaction of otopathogenic *P. aeruginosa* with macrophages has never been investigated. The results of the present study contribute to our understanding of the interaction between otopathogenic *P. aeruginosa* and primary macrophages.

Macrophages form an important line of host defense in innate immune system against infections (Aderem, 2003; Tam and Aderem, 2014; Zhang and Wang, 2014; Divangahi et al., 2015; Hume, 2015; Schultze et al., 2015). Some studies employ macrophage cell lines to understand the interaction of pathogens with immune cells that may not mimic the true characteristics of primary cells. These cell lines are fundamentally different from the primary cells in that they grow continuously in culture due to permanent alterations in their genes. Such changes could have an effect on the signaling cascades that are activated following interaction of immune cells with pathogens. The results of studies utilizing primary cells have been instrumental in developing our understanding regarding immune cell response to infection. Therefore, in the present study primary human MDMs and mouse BMMϕ have been used to investigate the interaction between otopathogenic *P. aeruginosa* and macrophages. This study for the first time demonstrated that otopathogenic *P. aeruginosa* enters and survives inside primary human MDMs and mouse BMMϕ in a dose and time dependent manner in context of ear infections. We also observed that serum opsonization has no significant effect on invasion of macrophages by *P. aeruginosa*. Since opsonization of pathogens by serum components facilitate phagocytosis through Fc gamma receptors on macrophages, these results suggest that otopathogenic *P. aeruginosa* employs unique mechanisms to enter inside macrophages. SEM demonstrated that otopathogenic *P. aeruginosa* adheres to the primary macrophages through the formation of pseudopod like structures. TEM confirmed the internalization of bacteria inside human MDMs and mouse BMMϕ. We also observed that survival of otopathogenic *P. aeruginosa* inside depends on the expression of OprF, the most abundant bacterial OMP/porin. On par with these findings, pretreatment of bacteria with anti-OprF monoclonal antibody significantly decreased the invasion of macrophages by *P. aeruginosa*, highlighting the crucial role of OprF in cell invasion. The ability of otopathogenic *P. aeruginosa* to survive inside macrophages provides protection against complement, lysozyme activity and other host immune defenses. This enables *P. aeruginosa* to evade killing and hence may constitute an important virulence trait.

Macrophages are a preferred niche for certain pathogens as they are highly phagocytic and long lived, providing protection for a prolonged period. Pathogens including *Candida glabrat*a, *Enterococcus faecalis, Brucella* sp., *Mycobacterium tuberculosis, Escherichia coli* and Group B streptococci, have also been demonstrated to enter and survive inside macrophages, which has been correlated with their ability to cause infection (Valentin-Weigand et al., 1996; Cornacchione et al., 1998; Pieters and Gatfield, 2002; Celli, 2006; Miramón et al., 2013; Miskinyte and Gordo, 2013; Elliott et al., 2015; Kasper et al., 2015; Sabatino et al., 2015). Other pathogens such as *Yersinia* spp. avoid uptake and phagocytosis preventing activation of macrophages thus avoiding potent host immune responses (Fällman et al., 2002). It has been demonstrated that tyrosine phosphorylation plays a crucial role in phagocytosis and subsequent activation of professional phagocytes (Park et al., 2011). However, pathogens produce effector proteins to neutralize tyrosine phosphorylation that promotes inhibition of phagocytosis and activation of macrophages. The effector protein, YopH, of *Yersinia* spp. possess tyrosine phosphatase activity thus counteracting the activating tyrosine phosphorylation signals of the host cell (Yuan et al., 2005). This protein is delivered into host cells through bacterial type III secretion system (T3SS) and prevents *Yersinia* internalization by macrophages by dephosphorylating the adaptor protein p130Cas, among other targets (Andersson et al., 1996). *Salmonella typhimurium* secretes the tyrosine phosphatase SptP, which is also injected into host cells through a T3SS (Kaniga et al., 1996). SptP deactivates Rho-family GTPases and subsequently abrogates activation of macrophages. In addition, in case of adherent *S. aureus*, it has been demonstrated that surface of biomaterial plays a crucial role in bacterial phagocytosis by macrophages (Domingues et al., 2015). *S. aureus* adhered to hydrophilic surfaces had a lowest rate of phagocytosis while bacteria adhered to common biomaterials such as silicone rubber, tissue culture polystyrene and stainless steel has intermediate rate of phagocytosis by J774A macrophage cell line. It was concluded that hydrophobicity is a necessary surface condition for effective phagocytosis of *S. aureus* by J774A macrophage cell line. Our previous studies have demonstrated that *P. aeruginosa* can invade human middle ear epithelial cells (HMEECs) (Mittal et al., 2014). However, the interaction of otopathogenic *P. aeruginosa* with primary macrophages has never been explored in previous studies.

Pathogens including *Shigella dysenteriae* utilize cytoskeletal rearrangement in order to gain entry inside host cells (Rottner et al., 2005). However, each pathogen utilizes unique mechanism to invade immune cells. We observed that internalization of *P. aeruginosa* inside human MDM and mouse BMMϕ relies on both microfilament and microtubule dependent uptake mechanisms. Pretreatment of human MDMs and mouse BMMϕ with actin polymerization inhibitors or microtubule-destabilizing agents led to a significant decrease in invasion of macrophages by *P. aeruginosa* in a dose-dependent manner. Some pathogens utilize only microfilaments whereas others employ microfilaments to gain entry inside host cells that are also cell type specific (Oelschlaeger et al., 1993; Kopecko et al., 2001; Mazon Moya et al., 2014; Valencia-Gallardo et al., 2015). However, the entry of other pathogens requires both microfilament and microtubule dependent uptake mechanisms (Ferrero et al., 2009; Taylor et al., 2010) as observed for otopathogenic *P. aeruginosa* in the present study.

Pathogens employ various strategies in order to survive inside the host and cause infection. Some pathogens trigger anti-apoptotic mechanisms to prevent host cell death. Pathogens including *Enterococcus faecalis, Toxoplasma gondii, Brucella* sp., *Neisseria meningitidis*, and *Neisseria gonorrhoeae* inhibit apoptosis of host cells, providing a niche where they can survive and replicate (Beck and Meyer, 2000; Tunbridge et al., 2006; Cai et al., 2014; Cui et al., 2014; Zou and Shankar, 2014). On the contrary, other microbes including *Mycobacterium tuberculosis, Legionella pneumophila, Bordetella pertussis, Listeria monocytogenes, Corynebacterium diphtheriae, Shigella flexneri*, and *Salmonella typhimurium*, induces cell death that allow the pathogens to efficiently exit the host cell, spread to neighboring cells, evade immune cells, and/or to gain nutrients (Haimovich and Venkatesan, 2006; Hewlett et al., 2006; Cervantes et al., 2008; dos Santos et al., 2010; Morinaga et al., 2010; Welin et al., 2011; Ashida et al., 2014; Behnsen et al., 2015). Thus, knowledge of the mechanisms employed by pathogens in the progression of disease is critical to have a better understanding of virulence and host defense. In this study, we observed that otopathogenic *P. aeruginosa* induces death of human MDMs and mouse BMMϕ as determined by LDH release in the cell culture supernatants and ethidium homodimer-I staining. LDH is the most acceptable and reliable marker for determining host cell death (Kwon et al., 2015; Park et al., 2015; Lv et al., 2016)^.^ Ethidium homodimer-I is a fluorescent nuclear stain that penetrates dead cells due to disrupted plasma membrane and increases intensity after binding to DNA (Eidet et al., 2015; McCanna et al., 2015; Yoeruek et al., 2016). The ability of otopathogenic *P. aeruginosa* to induce cell death in macrophages enables this pathogen to escape potent host defenses and will enable it to cause infection.

In summary, the results presented here demonstrate for the first time that otopathogenic *P. aeruginosa* are able to enter and survive inside macrophages in context of ear infections. Studies are in progress in our laboratory to elucidate the molecular mechanisms through which otopathogenic *P. aeruginosa* escape from macrophage killing. The uptake of otopathogenic *P. aeruginosa* by human MDMs and mouse BMMϕ relies on actin polymerization and microtubule dependent process. Bacterial OprF expression plays a crucial role in the intracellular survival of *P. aeruginosa* inside human MDMs and mouse BMMϕ. Future studies comparing the level of *oprF* gene expression between strains isolated from CSOM patients and the other diseases such as pneumonia or urinary infection will help in confirming the role of bacterial *oprF* for the establishment of CSOM. In addition, it has been demonstrated that pathogens form biofilms on middle ear during CSOM (Saunders J.E. et al., 2011; Kaya et al., 2013; Gu et al., 2014). Therefore, future investigations are warranted to determine the interaction of biofilm cells of otopathogenic *P. aeruginosa* with primary macrophages. Our findings suggest that otopathogenic *P. aeruginosa* is recognized by phagocytic cells but remain impermeable to attacks by their antimicrobial components and is able to exert cytotoxic effects on macrophages. Further studies employing mouse model of CSOM are warranted to delineate the role of macrophages in the disease process. Understanding host–pathogen interactions will provide novel avenues to design effective treatment modalities against CSOM, and hence, prevent consequent hearing loss as well as life-threatening CNS complications.

## Author Contributions

RM, CL, HK, MG, CJ, and PB performed the experiments. RM, KM, MG, and PB wrote the manuscript. RM, KM, PW, CJ, DY, and XL designed and supervised the study. All authors approved the final version of the manuscript.

## Conflict of Interest Statement

The authors declare that the research was conducted in the absence of any commercial or financial relationships that could be construed as a potential conflict of interest.
