# Supplementary material for: Otopathogenic Pseudomonas aeruginosa Enters and Survives Inside Macrophages
Source: Front Microbiol. 2016 Nov 18;7:1828. doi: 10.3389/fmicb.2016.01828 (PMC5114284; doi:10.3389/fmicb.2016.01828)
Supplement: Supplementary file 1 [file Data_Sheet_1.PDF]

Supplementary Figure Legends

Supplementary Figure 1. Otopathogenic *P. aeruginosa* invades mouse BMMϕ in a time and dose dependent manner. Mouse BMMϕ were infected with clinical isolate of *P. aeruginosa* at various MOI for 2h and intracellular survival was determined by gentamicin protection assay (a). In separate experiments, cells were infected with *P. aeruginosa* at different MOI for varying time periods and phagocytosis was determined (b). Data represents mean ± SD and is representative of five individual experiments carried out in triplicate. * $P<0.01$ or # $P>0.05$ by Student's *t* test and ANOVA.

Supplementary Figure 2. Phagocytosis of otopathogenic *P. aeruginosa* by human MDMs. Macrophages were infected with different strains of otopathogenic *P. aeruginosa* at an MOI of 10 for varying time periods and survival of bacteria was assessed by gentamicin protection assay. Data represents mean ± SD. Results are representative of five independent experiments carried out in triplicate.

Supplementary Figure 3. Otopathogenic *P. aeruginosa* are internalized by mouse BMMϕ. Cells were infected with *P. aeruginosa* at an MOI of 10 for 2, 4 and 6 h. The internalization of bacteria was determined by gentamicin protection assay. Data represents mean ± SD and is representative of four individual experiments carried out in triplicate.

Supplementary Figure 4. Scanning electron micrographs of mouse BMMϕ infected with *P. aeruginosa*. Mouse BMMϕ were infected with *P. aeruginosa* for 15 min (a), 30 min (b), 1h (c), 2h (d), 4h (e) and 8h (f) and subjected to SEM. Yellow arrows indicate pseudopod like structures and green arrows indicate bacteria. Results are representative of three individual experiments. Scale bars

Supplementary Figure 5. Transmission electron micrograph of mouse BMMϕ infected with *P. aeruginosa* demonstrating bacterial internalization and survival. Mouse BMMϕ were infected with *P. aeruginosa* for 15 min (a), 30 min (b), 1h (c), 2h (d), 4h (e) and 8h (f) and subjected to TEM. Yellow arrows indicate pseudopod like structures and green arrows indicate bacteria. Results are representative of three individual experiments. Scale bars

Supplementary Figure 6. *P. aeruginosa* induces cell death in macrophages. Human MDMs (a) and mouse BMMϕ (b) were infected with *P. aeruginosa* for varying time periods and macrophage cell viability was assessed by ethidium homodimer-1 staining. Results were expressed in percentage dead cells. Data represents mean ± SD and is representative of three individual experiments carried out in triplicate. *P<0.01 compared to WT or pOprF by Student's *t* test and ANOVA.

Supplementary Materials and Methods

Generation of OprF mutant and complementation

An unmarked *oprF* null mutant of *P. aeruginosa* was generated by gene splicing using primers *oprF* UF1 and *oprF* UR1 (ggaattgtgagcggataacaatttcacacaggaaacagctCACTTGAATAAGCCTCACCC and CCTTAGAGGCTCAGCCGATTActagttagctagCATCCGTTAAATCCCCATCT, respectively) to generate the upstream product P1, flanked by *Eco*RI and *Nhe*I sites and *oprF* DF1 and *oprF* DR2 primers (AGATGGGGATTTAACGGATGctagctaactagTAATCGGCTGAGCCTCTAAGG and ccaggcaaattctgttttatcagaccgcttctgcgttctgatTGGTCGACGATCTGGTCA) to generate the downstream product flanked by *Nhe*I and *Bam*H1 sites. After sequencing to ensure absence of mutations, P1 and P2 were spliced together to obtain a 1520 bp deletion fragment of *oprF* containing stop codons in all three reading frames at their junction (inserted as part of the *Nhe*I sites in the primer). This was then sequenced and subcloned into plasmid pEXG2 (Rietsch et al., 2005) as an *Eco*RI-*Bam*HI fragment and moved into *P. aeruginosa* strains by allelic exchange (Hmelo et al., 2015; Lehman et al., 2016; Schweizer and Hoang, 1995) using pRK600 (Kessler et al., 1992; Ramos et al., 1998; Balasubramanian et al., 2012) as the helper plasmid. Double crossover mutants were selected for the loss of plasmid (gentamicin-sensitive, sucrose counter-selection). The absence of *ΔoprF* was confirmed using PCR (Woodruff and Hancock, 1989; Hmelo et al., 2015) and restriction analysis of amplicons. For complementation, functional *oprF* gene from *P. aeruginosa* was cloned into pUCP19 (Woodruff and Hancock, 1989) by Genscript company (Piscataway, NJ) that imparts carbenicillin resistance. *oprF* from *P. aeruginosa* PAO1 was amplified using the primer pairs CCGTCTACTGCTCGATCAGC and ATTGGACCTGGACGGATTGA and then cloned into pUCP19 as described in detail in earlier studies (Woodruff and Hancock, 1989). The plasmid was moved into *ΔoprF P. aeruginosa* by

electroporation and expression was confirmed using Western blotting (Choi et al., 2006). *ΔoprF* and complemented strains were generated from three *P. aeruginosa* isolates.

References

Balasubramanian, D., Schneper, L., Merighi, M., Smith, R., Narasimhan, G., Lory, S., et al. (2012). The regulatory repertoire of *Pseudomonas aeruginosa* AmpC ß-lactamase regulator AmpR includes virulence genes. PLoS One. 7, e34067.

Choi, K.H., Kumar, A., Schweizer, H.P. (2006). A 10-min method for preparation of highly electrocompetent *Pseudomonas aeruginosa* cells: application for DNA fragment transfer between chromosomes and plasmid transformation. *J Microbiol Methods.* 64, 391-397.

Hmelo, L.R., Borlee, B.R., Almblad, H., Love, M.E., Randall, T.E., Tseng, B.S. (2015). Precision-engineering the *Pseudomonas aeruginosa* genome with two-step allelic exchange. *Nat Protoc.* 10, 1820-41.

Kessler, B., de Lorenzo, V., Timmis, K.N. (1992) A general system to integrate lacZ fusions into the chromosomes of gram-negative eubacteria: regulation of the Pm promoter of the TOL plasmid studied with all controlling elements in monocopy. Mol Gen Genet. 233, 293-301.

Lehman, M.K., Bose, J.L., Bayles, K.W. Allelic Exchange. *Methods Mol Biol.* 2016, 1373:89-96.

Ramos, J.L., Duque, E., Godoy, P., Segura, A. (1998) Efflux pumps involved in toluene tolerance in *Pseudomonas putida* DOT-T1E. *J Bacteriol.* 180, 3323-3239.

Rietsch, A., Vallet-Gely, I., Dove, S.L., and Mekalanos, J.J. (2005). ExsE, a secreted regulator of type III secretion genes in *Pseudomonas aeruginosa*. Proc. Natl. Acad. Sci. USA. 102, 8006-8011.

Schweizer, H.P., Hoang, T.T. (1995) An improved system for gene replacement and xylE fusion analysis in *Pseudomonas aeruginosa*. *Gene* 158, 15–22.

Woodruff, W. A., and Hancock. R. E. (1989). *Pseudomonas aeruginosa* outer membrane protein F: structural role and relationship to the *Escherichia coli* OmpA protein. *J. Bacteriol.* 171, 3304-3309.
