# Supplementary figures and images for: Otopathogenic Pseudomonas aeruginosa Enters and Survives Inside Macrophages
Source: Front Microbiol. 2016 Nov 18;7:1828. doi: 10.3389/fmicb.2016.01828 (PMC5114284; doi:10.3389/fmicb.2016.01828)

Supplementary Figure 1

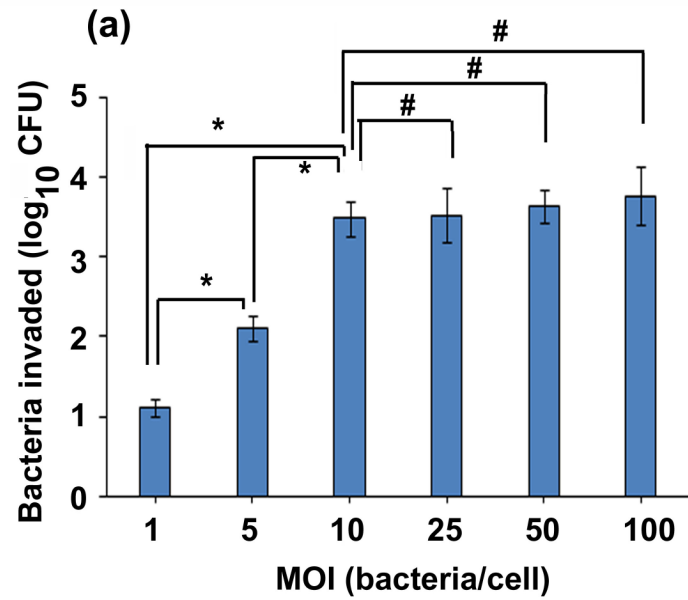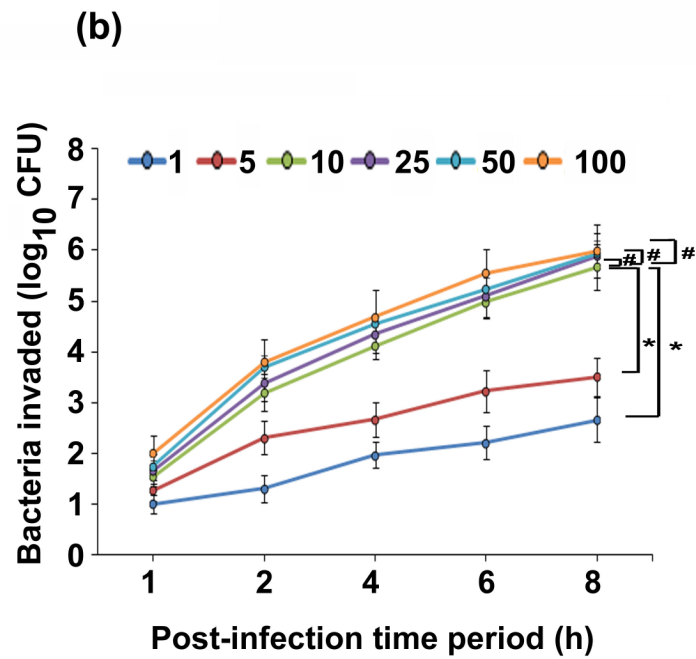

Supplementary figure 2

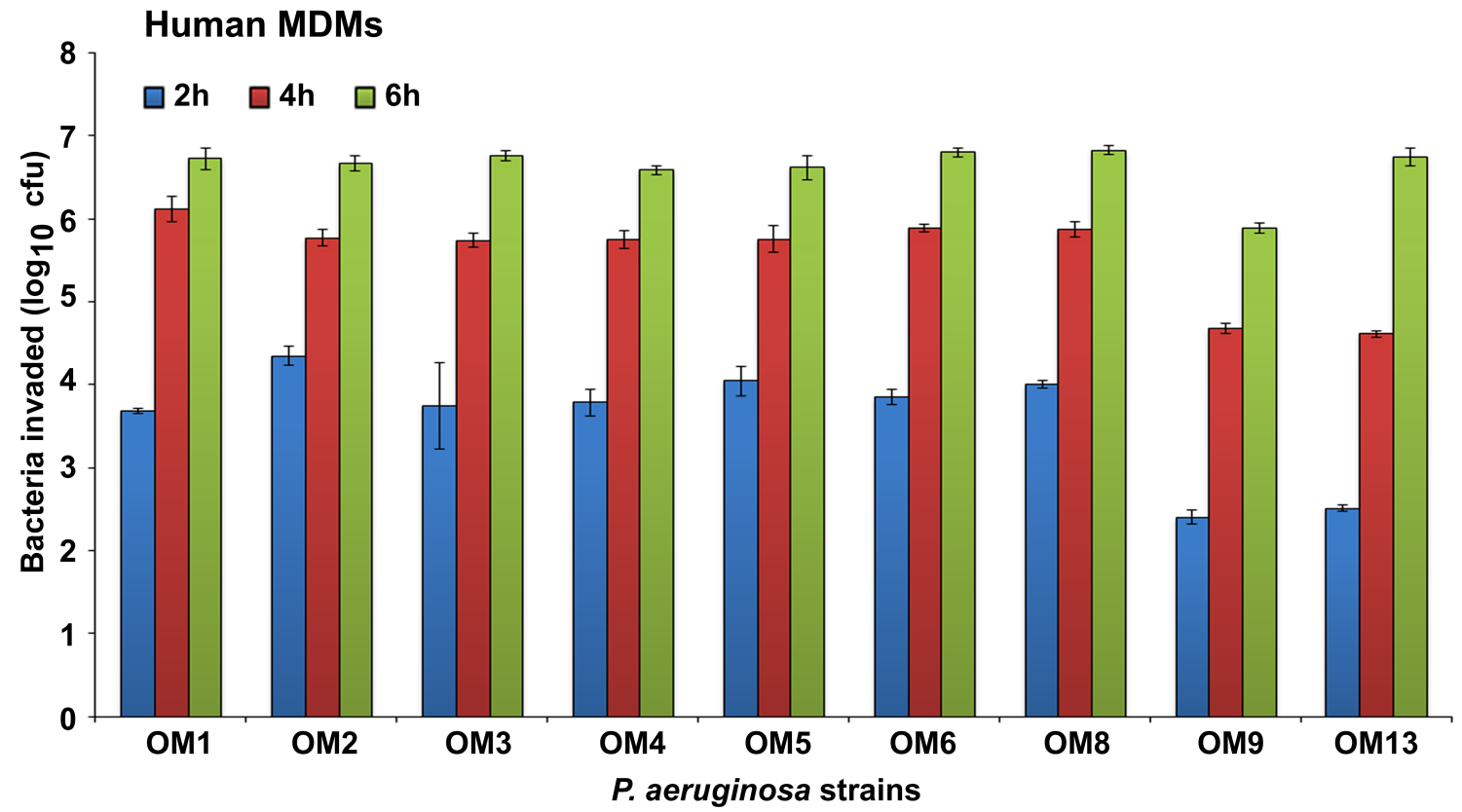

Supplementary Figure 3

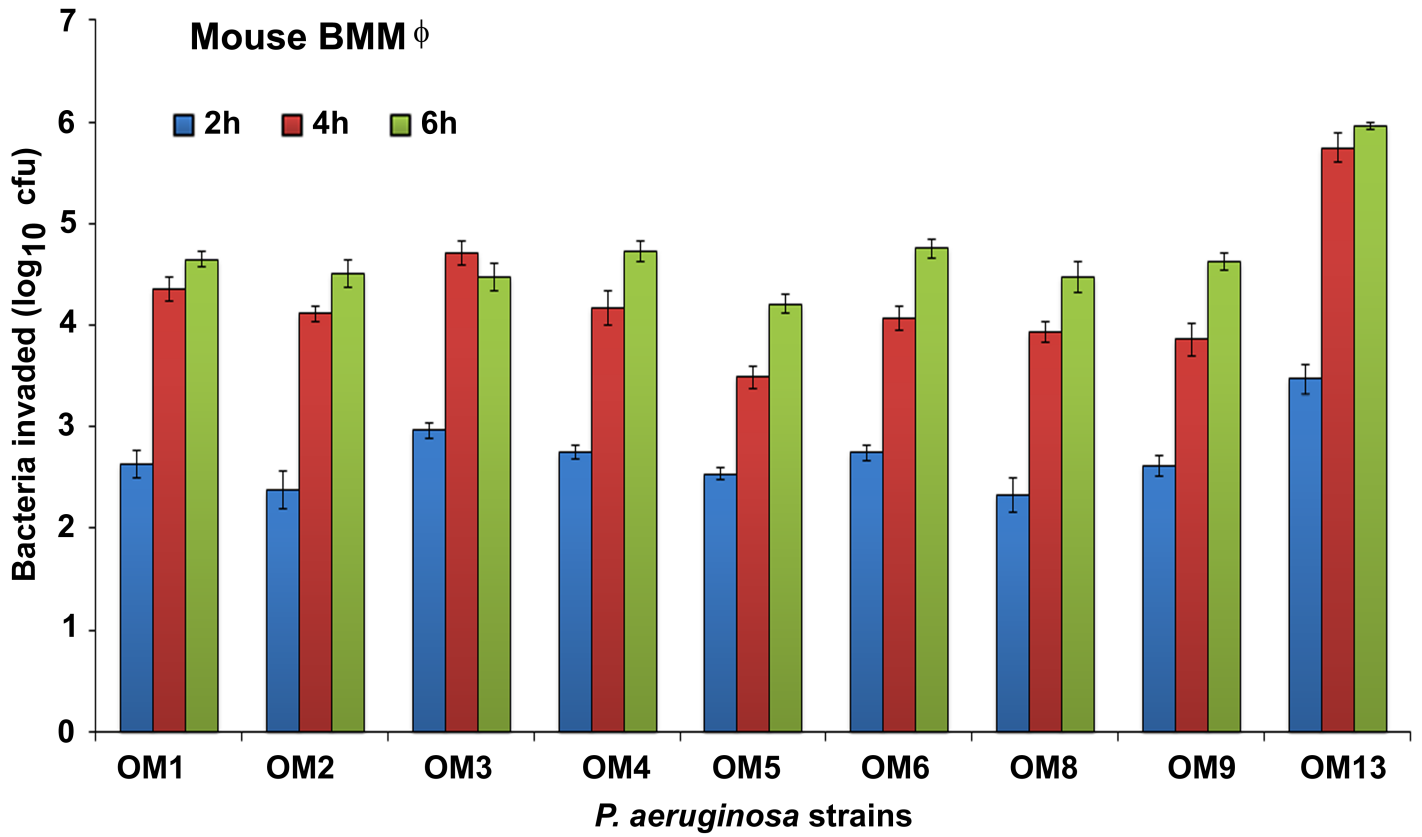

Supplementary Figure 4

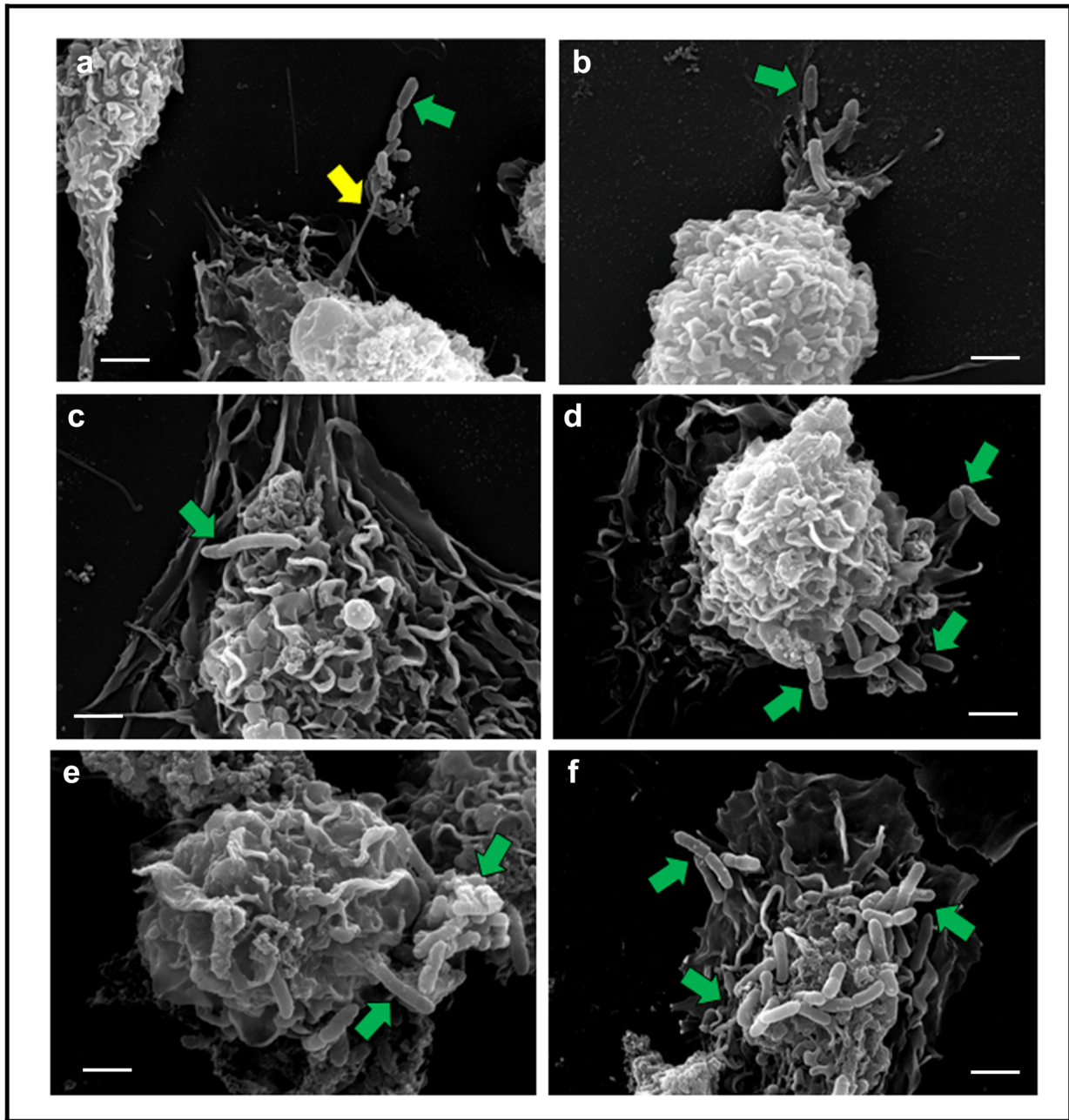

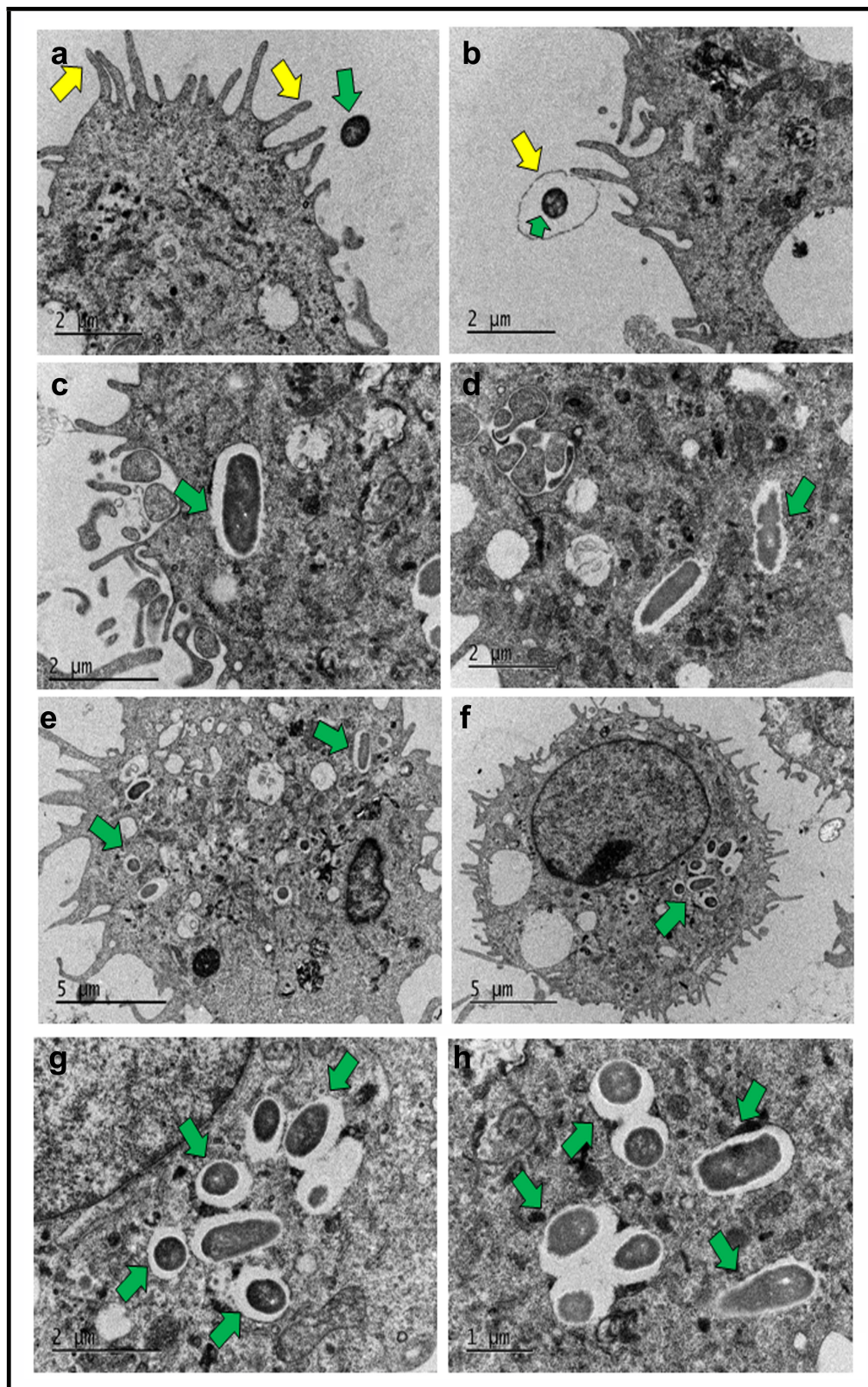

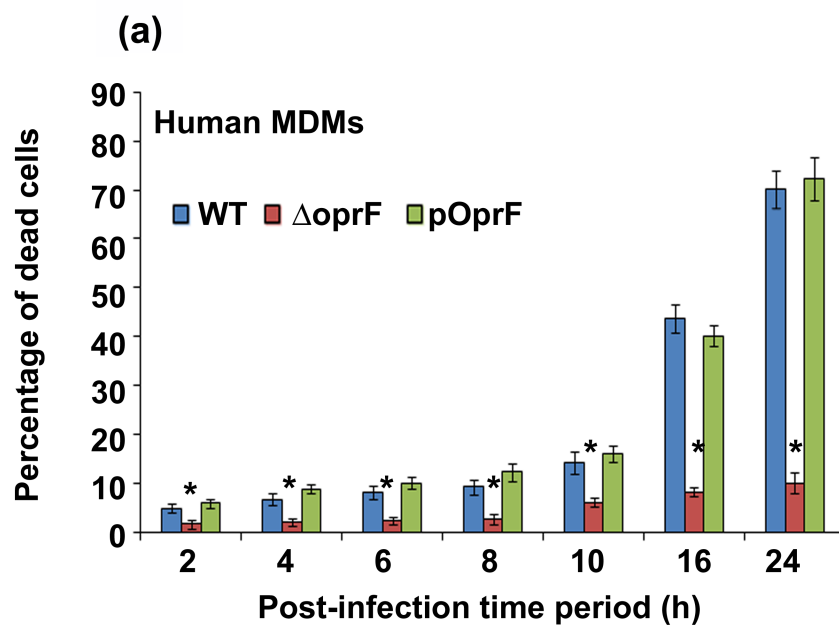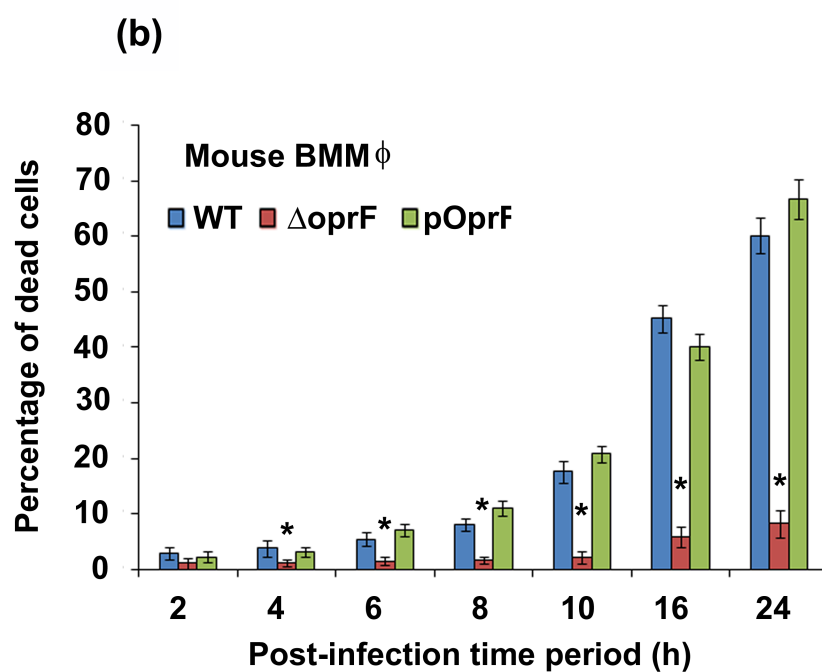

Supplement: Supplementary file 2 [file Image_1.PDF]
